# Phlobaphenes modify pericarp thickness in maize and accumulation of the fumonisin mycotoxins

**DOI:** 10.1038/s41598-020-58341-8

**Published:** 2020-01-29

**Authors:** Michela Landoni, Daniel Puglisi, Elena Cassani, Giulia Borlini, Gloria Brunoldi, Camilla Comaschi, Roberto Pilu

**Affiliations:** 1 Dipartimento di Bioscienze, Università degli Studi di Milano, Via Celoria 26, 20133 Milano, Italy; 2 Dipartimento di Scienze Agrarie e Ambientali - Produzione, Territorio, Agroenergia, Università degli Studi di Milano, Via Celoria 2, 20133 Milano, Italy

**Keywords:** Agricultural genetics, Plant breeding, Plant genetics

## Abstract

Phlobaphenes are insoluble phenolic compounds which are accumulated in a limited number of tissues such as seed pericarp and cob glumes, conferring on them a typical red-brown pigmentation. These secondary metabolites, derived from 3-deoxy flavonoids, are thought to have an important role in plants’ resistance against various pathogens, e.g. by reducing fungal infection, and also to have beneficial effects on human and animal health due to their high antioxidant power. The aim of this work was to determine the role of phlobaphenes in reducing mycotoxin contamination on maize kernels. We analysed the effect of the *P1* (pericarp color 1) gene on phlobaphenes accumulation, pericarp thickness and fumonisins accumulation. Analysing fumonisins accumulation in different genetic backgrounds through three seasons, we found a clear decrease of these toxins through the three years (Wilcoxon test, Z = 2.2, p = 0.0277) in coloured lines compared with the isogenic non-coloured ones. The coloured lines, carrying *P1* allele showed an increase of phlobaphenes (about 10–14 fold) with respect to colourless lines. Furthermore there was a correlation between phlobaphenes accumulation and pericarp thickness (R = 0.9318; p = 0.0067). Taken together, these results suggest that the *P1* gene plays a central role in regulating phlobaphenes accumulation in maize kernels, and indirectly, also tackles mycotoxins accumulation. The development and cultivation of corn varieties rich in phlobaphenes could be a powerful tool to reduce the loss of both quality and yield due to mycotoxin contamination, increasing the safety and the quality of the maize product.

## Introduction

The growing interest in foods rich in flavonoids and other bioactive molecules, regular consumption of which is associated with a reduced risk of chronic diseases^1–4^, has brought about a rediscovery of the importance of pigmented maize varieties. In particular the geneticist’s attention has focused on ancient varieties and landraces to find valuable genes for developing functional foods with high amounts of antioxidant compounds. It is known that ancient farmers’ varieties and landraces possess a broad natural variation in valuable nutraceuticals, but this has been impoverished during the breeding process which has led to modern cultivar development^5,6^.

Phenolic compounds, and in particular anthocyanins and phlobaphenes exhibit a noticeable antioxidant activity and confer a red, blue or black pigmentation to the plant tissues^7,8^.

Flavonoid biosynthesis is a complex pathway made up of more than 20 structural genes and it is regulated by two classes of transcription factors, the bHLH genes *(r1/b1*) and the *c1*/*pl1*/*p1* Myb genes families. This pathway is divided into two branches: one, regulated by the MYB genes *c1* and *pl1* leads to flavonols and anthocyanins synthesis and the other, regulated by the *p1* gene leads to phlobaphenes and maysin production.

Phlobaphenes are reddish insoluble pigments. Their biosynthetic pathway begins with the condensation of malonyl-CoA with p-coumaroyl-CoA, catalysed by the enzyme chalcone synthase (CHS) (encoded by the colorless2 locus, *c2*). The chalcone isomerase (CHI) enzyme converts the resulting naringenin chalcone into the flavanone naringenin that is converted to apiforol and luteoforol by the enzymes dihydroflavonol reductase-DFR (encoded by the *A1* gene) and the flavanone-3-hydroxylase-F3-H (encoded by the *Pr1* gene). Apiforol and luteoforol are then polymerized into phlobaphenes^2,9–11^.

The accumulation of phlobaphene pigments in the maize pericarp layer is regulated by the R2R3-MYB transcription factor PERICARP COLOR1 (*P1*), with different *P1* alleles conferring different pericarp and cob glume colors^2,12^. The *P1-rr* allele determines the coloration of both pericarp and cob glumes: with the P*1-rw* allele only the pericarp is colored, with *P1-wr* only the cob glumes, and with *P1-ww* both the tissues are colorless^2,13,14^.

Preliminary results indicated that phlobaphene pigments could be associated with a reduced level of mycotoxin contamination, in particular a reduction in fumonisin B1, in maize kernels, as reported in our previous paper^15^.

The contamination of maize kernels by fumonisins is due to *Fusarium* infection, in particular by *F. verticillioides* and *F. proliferatum*. *Fusarium* hyphae have been reported to spread within the pericarp, colonizing the kernel in a radial pattern: the starburst symptoms of the colonization appear subsequently, in correspondence with the extensive dissolution of the pericarp walls^16^.

Fumonisins contamination is implicated in various serious animal diseases such as porcine pulmonary edema, equine leukoencephalomalacia, hepatotoxicity and carcinogenicity in rats^17–20^, and seems to be related to human oesophageal cancer^21^. In fact, even if, to date, a causal relationship between fumonisins exposure and human cancer has not been demonstrated, epidemiological studies in China, Iran and South Africa showed higher risk of oesophageal cancer in areas with higher exposure to fumonisins^22^, and the International Agency for Research on Cancer (IARC) classified FB1 as a possible carcinogen for humans (group 2B).

To reduce fumonisins exposure, several official agencies such as the FAO/WHO Expert Committee on Food Additives, U.S. Food and Drug Administration and the European Union, have established maximum levels of fumonisin content in non-processed maize (4 ppm), and maize meal (2 ppm) for human consumption.

The chronic exposure to mycotoxins represents a critical factor for human health, especially in the case of malnourished populations in low income countries^23^. In fact in rural subsistence regions of Southern Africa, where maize is the major staple crop, even when the level of mycotoxin contamination is under the maximum level, the exposure to mycotoxin can exceed the safe levels because of the undiversified maize-based diet. Maize cultivated in these areas is often prone to pre- and post-harvest mycotoxin contamination because of poor agricultural practices such as sowing untreated seeds from the previous season, late planting, incomplete drying of grains, or long storage of crops in facilities without adequate aeration, moisture and temperature control^23^.

Important results on the reduction of pre-harvest fumonisins contamination came from genomic assisted breeding programs, that allowed the identification of QTL and candidate genes for the selection of resistant maize lines^24^, and the development of the transgenic *Bt* maize, where the expression of the insecticidal Cry protein from *Bacillus thuringensis* allows the reduction of insect damage, *Fusarium* infection and fumonisins accumulation^25^.

Crop growing techniques can reduce the risk of *Fusarium* infection but there are no definitive strategies to prevent fungal infection and fumonisins accumulation in maize kernels. In 2017 in Italy about 37% of the sample analysed showed a level of fumonisins contamination higher than the legal limit^26^.

The selection of varieties rich in phlobaphenes and other flavonoids sharing with them a part of the biosynthetic pathway, could thus represent an interesting opportunity, not only because of their beneficial effects on human health due to their antioxidant activity, but also because of their role in protecting maize plants from fungal infections, reducing mycotoxin contamination, thus increasing the quality of the kernel and its healthiness.

The maize pericarp has been shown to play an important role in resistance against *Fusarium* attacks, as demonstrated by the inverse correlation between pericarp thickness and *Fusarium* attacks susceptibility^27^, and the identification of the pericarp and its wax layer as resistance factors to fumonisins accumulation^28^.

In this context the ancient cultivar Nero Spinoso from the Camonica Valley (Italy) appears very interesting. It is characterized by a very high level of phlobaphenes and a pericarp layer thickness which was significantly higher than that in the colourless control^29^.

The aim of this work was to better clarify previous preliminary data regarding the relationship between the presence of phlobaphenes in maize pericarp and mycotoxin content, using new genetic materials characterized by the presence of high levels of phlobaphenes, together with the isogenic colourless lines.

## Results

### Constitution of the genetic material and flavonoid analysis

**Figure 1:**
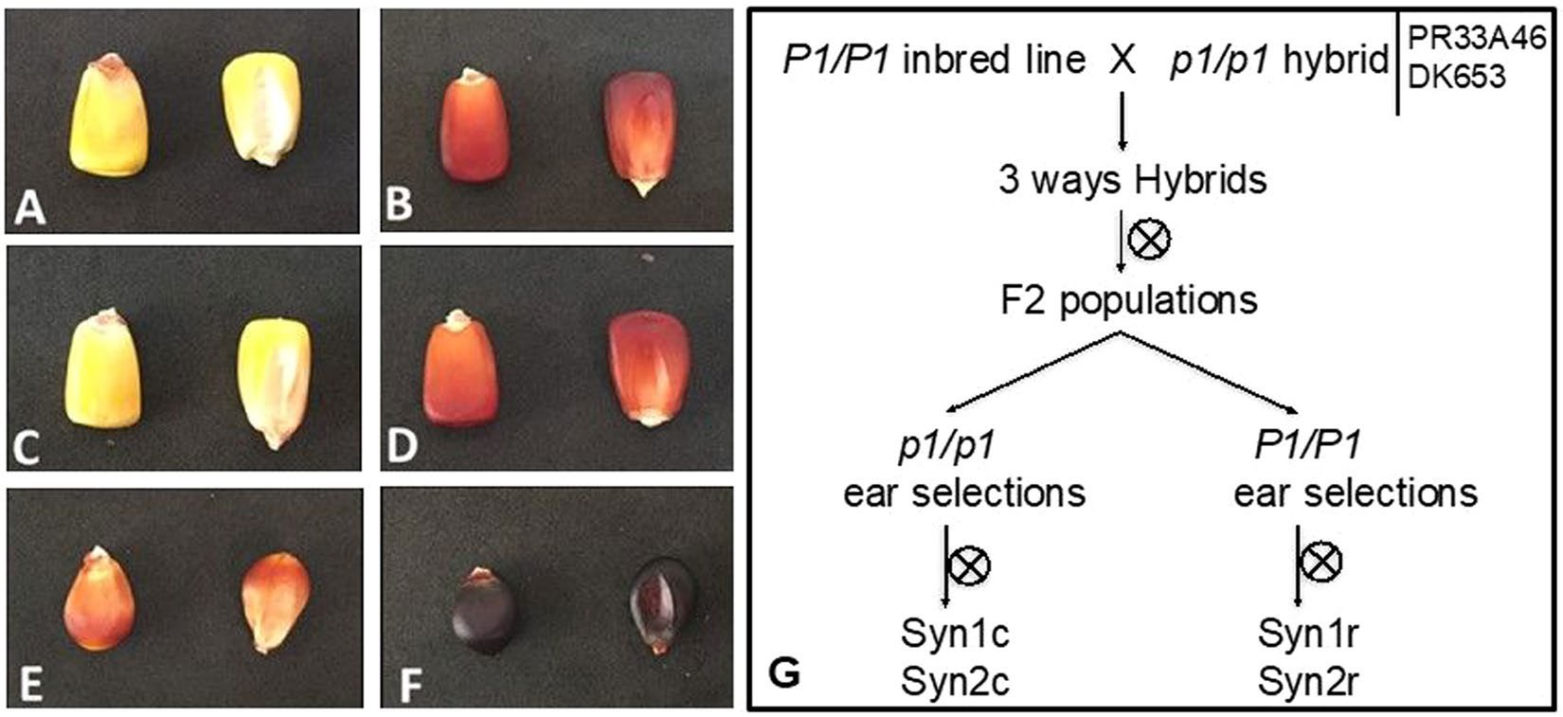
Mature seeds of the maize lines used in this work: Syn1c (**A**), Syn1r (**B**) Syn2c (**C**), Syn2r (**D**), Nsw (**E**), Nsr (**F**). Scheme of the crosses performed to obtain Syn populations (**G**).

With the aim of studying the relationship between the presence of the *P1* gene and the mycotoxin contamination on maize seeds, we produced as described in the Materials and Methods section, four synthetic populations, Syn1r and Syn2r characterized by the presence of the *P1* gene, and Syn1c and Syn2c the colorless *p1* controls. We also used two sub-populations of “Spinoso Nero di Esine della Val Camonica” characterized by a high level of phlobaphenes in the pericarp layer: NSr (fully pigmented ears) and NSw (weakly pigmented ears) (Fig. 1).

We cultivated these genetic materials for three years (2015–2016–2017) at the experimental field of the Faculty of Agricultural and Food Sciences of Milan. The materials were analyzed every year for the phlobaphenes, flavonols and phenolic acids amounts.

Higher amounts of phenolic compounds were found in *P1/P1* varieties compared with the *p1/p1* controls (Table 1). In particular, our analysis showed that the *P1* allele conferred a tenfold higher concentration of phlobaphenes in comparison with the colorless controls. In fact Syn1c (*p1/p1*) showed a phlobaphenes concentration of 13.44 (A510/100 g) and the isogenic colored Syn1r (*P1/P1*) had a concentration of 183.51 (A510/100 g). The phlobaphenes content in Syn2c (*p1*/*p1*) was 9.28 (A510/100 g) and in Syn2r (*P1/P1*) was 105.03 (A510/100 g). Similarly Nsw *(p1/p1)* had a phlobaphenes concentration of 46.43 (A510/100 g) and Nsr (*P1/P1*) of 350.24 (A510/100 g). The trend was maintained similarly for flavonols content, the colorless varieties Syn1c and Syn2c (*p1/p1*) showing an amount of 84.18 ± 6.26 mg/100 g and 72.59 ± 5.43 mg/100 g respectively, while in the colored isogenic hybrids Syn1r and Syn2r the flavonols content was respectively of 91.79 mg/100 g and 86.01 mg/100 g. Similar results were also obtained for phenolic acids content, colorless varieties Syn1c and Syn2c showed a concentration of 146 mg/100 g and 135 mg/100 g respectively while colored varieties, Syn1r and Syn2r had amounts of 165 mg/100 g and 158 mg/100 g. We also observed that the synthetic populations derived from the commercial hybrid PR 33A46, the colored Syn1r and the colorless Syn1c, had a higher amount of all phenolic compounds tested compared respectively with the colored Syn2r and the colorless Syn2c lines derived from commercial hybrid DK 6530 (Table 1). We noticed small differences between the same genotypes in the different seasons (Table 1), confirming the role of the *P1* gene as the main regulatory gene of the phlobaphenes pathway, and a small environmental effect as expected for a qualitative trait.

**Table 1:** Spectrophotometric analysis of phlobaphenes, flavonols and phenolic acids quantified respectively as absorbance at 510 nm, mg quercetin 3-glucoside equivalents and ferulic acid equivalents per 100 g of dry seed flour. SD are shown (n = 3).

Year	Code	Genotype	Phlobaphenes (A510/100 g)	Flavonols (mg/100 g)	Phenolic acids (mg/100 g)
2015	Syn1c	*p1*	13.44 ± 3.04^a^	84.18 ± 6.26^bc^	146 ± 11^ab^
	Syn1r	*P1*	183.51 ± 20.96^d^	91.79 ± 7.13^b^	165 ± 23^ab^
	Syn2c	*p1*	9.28 ± 1.29^a^	72.59 ± 5.43^c^	135 ± 16^b^
	Syn2r	*P1*	105.03 ± 9.93^c^	86.01 ± 5.75^b^	158 ± 18^ab^
	NSw	*p1*	46.43 ± 9.31^b^	101 ± 17.23^ab^	146 ± 22^ab^
	NSr	*P1*	350.24 ± 38.52^e^	152 ± 34.43^a^	170 ± 14^a^
2016	Syn1c	*p1*	11.12 ± 5.84^a^	76.34 ± 4.87^bc^	165 ± 21^a^
	Syn1r	*P1*	165.45 ± 16.93^d^	87.21 ± 4.80^b^	148 ± 26^a^
	Syn2c	*p1*	7.85 ± 2.23^a^	68.54 ± 7.45^c^	141 ± 23^a^
	Syn2r	*P1*	110.16 ± 12.72^c^	92.32 ± 8.36^b^	172 ± 27^a^
	NSw	*p1*	56.73 ± 13.55^b^	112 ± 34.54^abc^	157 ± 14^a^
	NSr	*P1*	353.78 ± 34.86^e^	168 ± 38.32^a^	156 ± 18^a^
2017	Syn1c	*p1*	12.64 ± 3.63^a^	89.72 ± 6.84^b^	138 ± 22^a^
	Syn1r	*P1*	187.54 ± 32.67^d^	94.83 ± 12.50^b^	161 ± 14^a^
	Syn2c	*p1*	11.31 ± 2.69^a^	66.73 ± 8.63^c^	142 ± 25^a^
	Syn2r	*P1*	112.32 ± 11.12^c^	89.75 ± 7.83^b^	163 ± 26^a^
	NSw	*p1*	54.65 ± 6.79^b^	117 ± 19.96^ab^	142 ± 54^a^
	NSr	*P1*	361.54 ± 43.84^e^	163 ± 27.48^a^	166 ± 19^a^

For each year, means followed by the same letter are not significantly different (Tukey test, p < 0.05). For each genotype, no statistical differences were noticed among 2015–2017 seasons.

### Fumonisins quantification and pericarp thickness

In order to establish a possible correlation between pigmentation and fumonisins contamination in maize seeds we cultivated in adjacent separated plots of 140 square meters, under the same agronomic conditions, the four synthetic populations (Syn1c/r and Syn2c/r) for three years (seasons 2015, 2016, 2017). We determined the total fumonisins accumulation by an immune-enzymatic assay for the quantitative analysis of total fumonisins. Data shown in Table 2 indicate that in each sample analyzed the amount of mycotoxins was higher in the colorless flour in comparison with the colored one. In particular Syn1r (*P1/P1*) compared with the equivalent isogenic colorless variety Syn1c (*p1/p1*) showed an average fumonisins decrease over the three years of trials of 39.2%. A decrease of 19% was also present in Syn2r (*P1/P1*) compared with the equivalent isogenic colorless Syn2c (*p1*/*p1*). Using a non-parametric Wilcoxon rank sum test to analyze these data we found statistically significant differences (Z = 2.2 p = 0.0277) for fumonisins content in colored and colorless materials (Table 2).

**Table 2:** Determination of fumonisins concentration in maize flour (ppb) using ELISA’S test.

	Genotype		Decrease (%)	Genotype		Decrease (%)
	Syn1c (*p1*)	Syn1r (*P1*)		Syn2c (*p1*)	Syn2r (*P1*)	
1° year	128.4 ± 14.7^a^	69.6 ± 10.2^b^	45.8	349.2 ± 40.7^a^	294.5 ± 47.5^a^	15.7
2° year	4520.4 ± 353.1^a^	2888.8 ± 246.7^b^	36.1	22176.8 ± 625.8^a^	17436.3 ± 563.6^a^	21.4
3° year	2171.9 ± 237.6^a^	1398.3 ± 157.3^b^	35.6	10654.4 ± 423.2^a^	8543.9 ± 273.8^a^	19.8
Average decrease (%)			39.2			19

Confidence interval at 95% are shown (n = 3). For each year, the means followed by the same letter are not significantly different, comparing Syn1c with Syn1r and Syn2c with Syn2r. The non-parametric Wilcoxon rank sum test revealed statistically significant differences (Z = 2.2, p = 0.0277) between colored and colorless materials.

**Figure 2:**
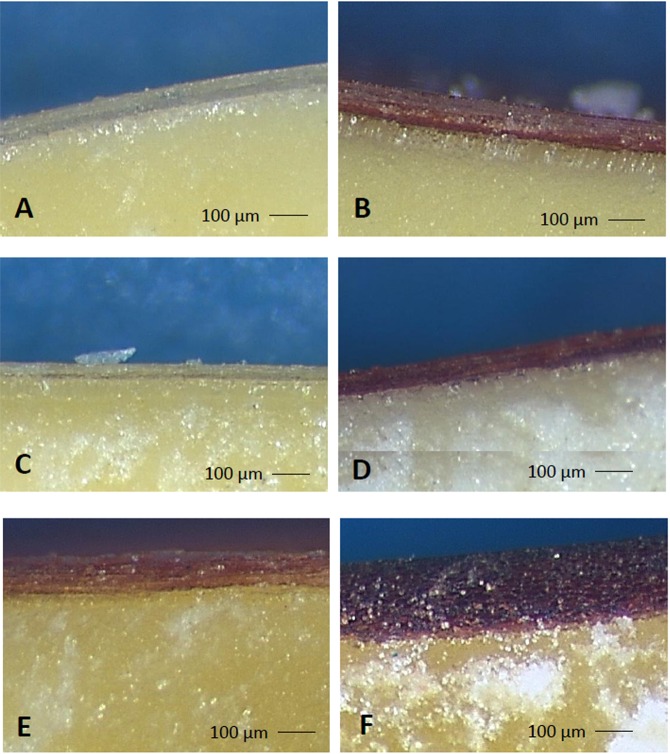
Histological analysis of mature seeds pericarp thickness: Syn1c (**A**), Syn1r (**B**), Syn2c (**C**), Syn2r (**D**), Nsw (**E**) and Nsr (**F**).

**Figure 3:**
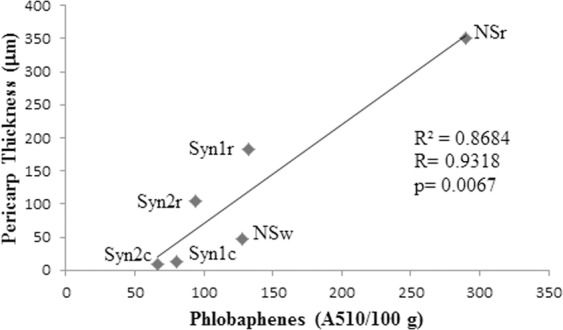
Correlation analysis between phlobaphenes amount and pericarp thickness of the seed in Syn1c, Syn1r, Syn2 c, Syn2r, Nsw and Nsr.

In order to study whether different levels of phlobaphenes were associated with differences in seed morphology we focused our attention on the pericarp, where these pigments are accumulated. The histological analysis showed differences in pericarp thickness among the different varieties, in agreement with the data concerning phlobaphenes quantification. In fact the colorless varieties (Syn1c = 80 µm; Syn2c = 66 µm; Nsw = 128 µm) had a thinner pericarp if compared with equivalent isogenic colored varieties (Syn1r = 132; Syn2r = 94 µm; Nsr = 290 µm) (Fig. 2 and Table 3). A strong correlation (R = 0.9318, p = 0.00679) was found between phlobaphenes content and pericarp thickness by analyzing the data collected (Fig. 3).

**Table 3:** Measurements of mature seeds pericarp thickness expressed as µm.

Code	Genotype	Pericarp thickness (µm)
Syn1c	*p1*	80 ± 7.07^ab^
Syn1r	*P1*	132 ± 4.47^c^
Syn2c	*p1*	66 ± 4.18^a^
Syn2r	*P1*	94 ± 9.61^b^
NSw	*p1*	128 ± 9.08^c^
NSr	*P1*	290 ± 15.81^d^

The analyses were carried out in the 2015 season. SD are shown (n > 15). Means followed by the same letter are not significantly different (Tukey test, p < 0.05).

### *P1* gene is responsible for increasing pericarp thickness

**Figure 4:**
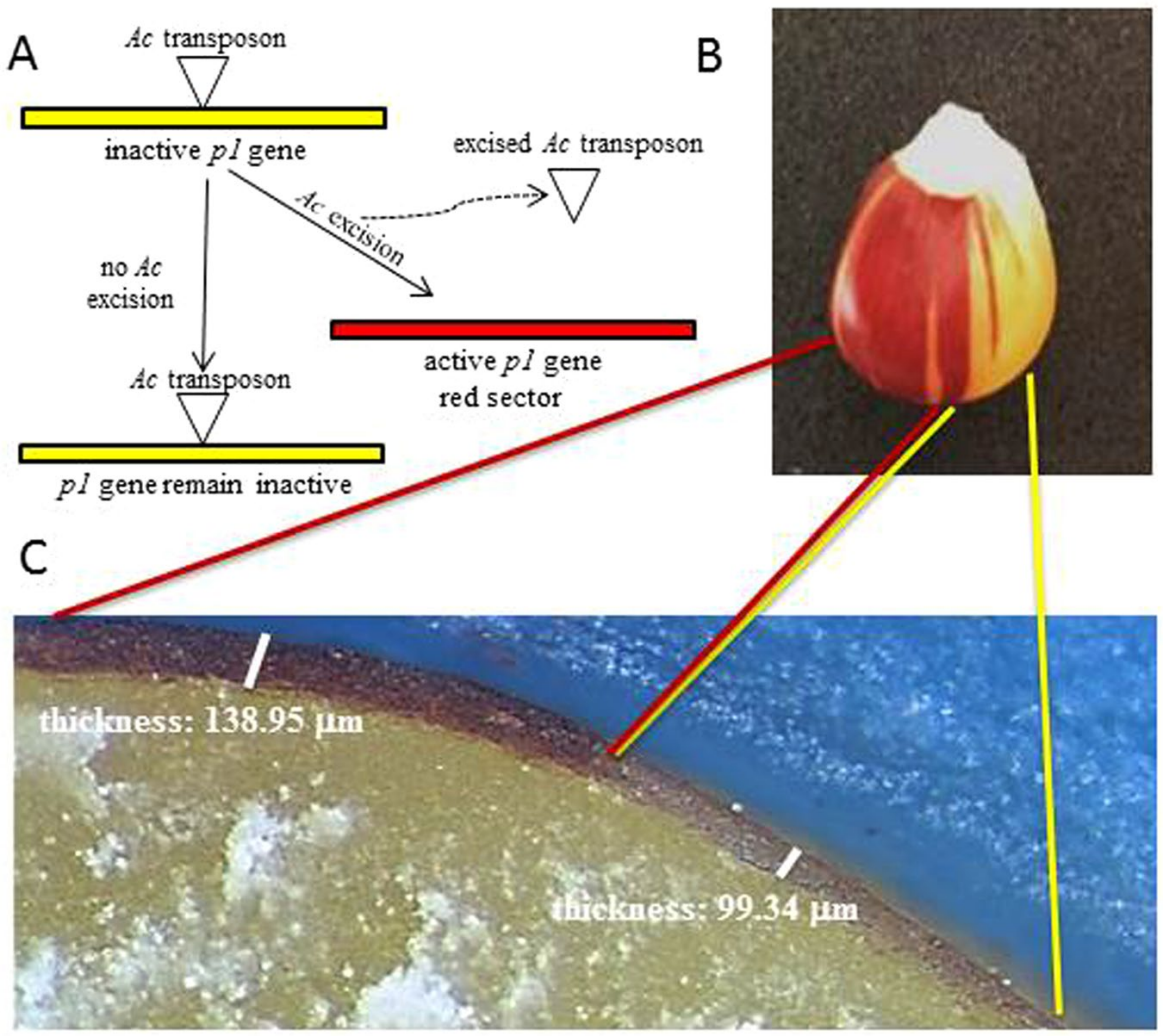
Scheme explaining how the *Ac* excision from *P1* gene produced somatic sectors due to the reactivation of phlobaphenes pathway (**A**). Seed sectors caused by *Ac* excision from the locus *p1* (*PVV* unstable allele). (**C**) Histological analysis of mature PVV seeds and pericarp thickness of colorless and red sectors.

With the aim of strengthening the idea that the *P1* gene is able in a specific way to modify the pericarp thickness, we measured colorless and colored sectors in PVV seeds. We used an inbred line characterized by a variegated pericarp color due to the excision of an Ac element in the *P1* locus (PVV system). Hence the only difference present in these sectors was due to the presence of the pigments/activity of *P1* gene, all the other genes were the same. As shown in Fig. 4, in the *p1* colorless sectors the pericarp thickness was of 99.34 ± 6.8 µm whilst in the *P1* colored sectors it was 138.95 ± 8.5 µm (n >15, SD are shown), i.e. the higher level of phlobaphenes is associated with higher pericarp thickness.

## Discussion

Phlobaphenes are reddish insoluble pigments derived from 3-deoxy flavonoids produced by a specific branch of the flavonoids pathway together with anthocyanins (derived from 3-hydroxy flavonoids)^4,11^. The biosynthetic pathway of phlobaphenes is regulated by the *P1* gene^30^, a transcription factor member of the MYB gene family. In maize they are accumulated in a limited number of tissues, such as seed pericarp, a tissue of maternal origin corresponding to the ovary wall, and cob glumes^29,31,32^, and confers on them a typical red-brown pigmentation which is sometimes very dark, as in the italian Nero Spinoso variety traditionally cultivated in the Camonica Valley^29^.

Preliminary data had shown the role of phlobaphenes and other flavonoids accumulated in maize pericarp on the reduction of fungal ear rot and fumonisins accumulation^15,33^. The phlobaphenes and other secondary metabolites are accumulated in many ancient landraces traditionally cultivated in the Padana Plane and in mountainous regions of Northern Italy^29,33^.

Fumonisins are mycotoxins produced by different species of *Fusarium* able to infect maize, especially *F. verticillioides* and *F. proliferatum*, and are associated with the development of various serious diseases in both humans and animals^18^. Correct cultural practices such as wet planting, removal of debris from previous crops, use of insect resistant maize varieties and a low level of nitrogen fertilizer, have been reported to reduce the spread of *Fusarium* infection and fumonisins content^34^ but the most effective strategies rely on genetic improvement of maize. In particular, due to the multigene nature of *Fusarium* resistance, a promising approach seems to be the development of maize lines with high phenolic compounds accumulated in the pericarp, which have proven to be more resistant to *Fusarium* infection and to have a reduced content of mycotoxins. The phenolic compounds have been reported to be involved in the reduction of the susceptibility of maize plants to *Fusarium* attack, acting as a physical barrier by hardening the maize pericarp^15,27^, or inactivating fungal proteins by the formation of irreversible complexes^35^, or targeting the fungal antioxidative stress response^36^.

With the aim of further dissecting the relationship between phlobaphenes accumulation and resistance to *Fusarium* infection we analyzed maize seeds from four different genetic backgrounds characterized by the presence of high levels of phlobaphenes in the pericarp and the corresponding colorless isogenic lines as controls (Syn1r, Syn1c, Syn2r and Syn2c).

The data showed that in the flour of colorless varieties, mycotoxins’ content was higher than in the colored ones (Table 2). In particular Syn1r (*P1/P1*) showed an average decrease of fumonisins content of 39.2% compared with the corresponding isogenic colorless population Syn1c (*p1/p1*). A similar decrease was observed for Syn2r (*P1/P1*) which showed an average decrease of 19% compared with the equivalent isogenic colorless population Syn2c (*p1/p1*). Our results are in agreement with previously reported data suggesting that phlobaphenes and other flavonoids are implicated in kernel resistance against fungal infection. In particular the decrease of fumonisins amounts in colored lines could be due to a direct effect of phlobaphenes that can form irreversible complexes with fungal proteins leading to their inactivation^35^ or to the barrier effect due to the increased pericarp thickness in colored lines in comparison to the colorless ones^37^. To check these hypotheses we quantified the phlobaphenes, flavonol and phenolic compound content and the pericarp thickness in each variety. The amount of phenolic compounds, despite small differences that can be explained by the variation of climatic conditions in the different agronomic seasons, was similar in every agronomic season analyzed, with an higher amount of phenolic compounds in colored lines (*P1/P1*) compared with the corresponding isogenic colorless (*p1/p1*) lines (Table 1). Focusing on phlobaphenes, we found that the *P1* allele was responsible for a more than 10 fold increase of these pigments in colored lines (Syn1r, Syn2r and Nsr) in comparison with the colorless controls (Syn1c, Syn2c, Nsw) (Table 1). In particular the population Nsr showed the highest level of pigments, responsible for the dark pigmentation of the seeds.

The histological analysis showed differences in pericarp thickness between colored and colorless lines. In particular the colorless lines (Syn1c = 80 µm; Syn2c = 66 µm; Nsw = 128 µm) had a thinner pericarp if compared with equivalent isogenic colored lines (Syn1r = 132 µm; Syn2r = 94 µm; Nsr = 290 µm) (Table 3, Fig. 2).

Taken together these data highlighted a strong correlation between pericarp thickness and phlobaphenes concentration (Fig. 3). The association between the phlobaphenes accumulation, driven by the *P1* gene, and the increase of pericarp thickness is also supported by the histological analysis of the *Ac* line. In this line in fact, the Ac element inserted in the *P1* gene confers variegated color and thickness to the pericarp. In these seeds, in the colorless *p1/p1* sectors the pericarp thickness is 99.34 µm while in colored *P1/P1* sectors the thickness is 138.95 µm (Fig. 4).

Considering the role of the *P1* gene in phlobaphenes accumulation and in pericarp thickness determination, these results suggested three different hypotheses: a) *P1* gene has a direct role in regulating pericarp thickness and color; b) pericarp thickness is a consequence of phlobaphenes accumulation driven by the *P1* gene; c) there is a linkage drag between *P1* gene, regulating phlobaphenes accumulation, and the gene “X” playing a central role in regulating the pericarp thickness. The histological characterization of the Ac line, seems to suggest a direct involvement of the *P1* gene in both pericarp color and thickness and thus seems to exclude the third hypothesis. The analysis of new genetic material will be necessary to collect evidence supporting one of the two alternative remaining hypotheses.

Our findings therefore suggest that the cultivation of maize varieties rich in phlobaphenes would enhance crop quantity and quality in areas which are characterized by humid and rainy climates particularly favorable to fungal development. In these varieties in fact the presence of phlobaphenes will ensure high resistance to fungal infection, and therefore a low level of mycotoxin contamination, together with the health promoting effect associated with this class of flavonoids. The beneficial properties derived from flavonoids have been well studied in recent years^2,4,38^ and although the effects of phlobaphenes on human and animal health are not yet completely known, their high antioxidant power suggests effects similar to those of the anthocyanins^1^.

Our work therefore highlighted the importance of the rediscovery of ancient pigmented varieties for use in breeding programs aiming to obtain a functional and safe food for human nutrition and animal feeding.

## Materials and Methods

### Plant material

In this work we used four synthetic populations, one Italian landrace, and one inbred line carrying the Ac transposon in *P1* gene (PVV) obtained as described below.

The four synthetic populations were obtained by crossing each of the two commercial hybrids PR 33A46 and DK 6530 with an inbred line carrying the *P1* gene which leads to phlobaphenes accumulation in the pericarp (*P1* line throughout the text).

The two F1s obtained were selfed, about 1000 seeds were sown in the following season and about 200 plants were selfed. The *p1/p1* control synthetic population was obtained by bulking the seeds coming from the colorless ears (segregation ratio ¾ colored: ¼ colorless).

The F3 colored ears (*P1/P1* and *P1/p1*) were selected for *P1/P1* genotype by testing the segregation of colored ears obtained from a sample of 20 seeds/ear. In this way we selected about 50 *P1/P1* ears that were shelled and the seeds obtained bulked to obtain the colored population. We named the synthetic populations coming from the PR33A46 parental Syn1r (red seeds) and Syn1c (colorless seeds), and the synthetic populations coming from the DK6530 Syn2r (red seeds) and Syn2c (colorless seeds).

We used the pigmented variety “Spinoso Nero di Esine della Val Camonica” which can accumulate in the pericarp layer a high level of phlobaphenes and the weakly pigmented sub-population present at the frequency of 2.87% in this colored variety^29^. We named these two sub populations NSr (pigmented ears) and NSw (weakly pigmented ears) throughout the text.

Finally we used an inbred line carrying the *Ac* autonomous component of the *Ac/Ds* system in the *P* locus, conferring a variegated pericarp due to transposon excision sectors (PVV system)^39^.

The four synthetic populations used for the determination of fumonisin amounts were tested in three field seasons (2015, 2016, 2017). For each population 1200 seeds were sown in adjacent separated plots of 140 square meters, under the same agronomic conditions, in the experimental field of the University of Milano located in Landriano (PV).

### Sample preparation and milling

About 70 ears of each variety/year were shelled and the seeds obtained mixed to create a single bulk used to perform various analyses. We milled the bulked seeds (about 10–15 Kg) by using an electric mill (Golia 4 V, Novital Italy) to obtain a coarse grinding. Flour samples were obtained using a ball mill (Retsch MM200, Retsch GmbH Germany), grinding for five min at 21 oscillations s^−1^ frequency, to a final size <20 mesh.

### Spectrophotometer determination of flavonols and phenolic acids

Fifteen mg of flour were boiled with 100 µl of distilled water for 30 minutes and then left in an overnight agitator with one ml of extraction buffer (1% HCl, 95% ethanol). After a further agitation of two hours with 500 µl of extraction buffer, the supernatants were collected together and centrifuged for 30 minutes. Their absorbance was determined spectrophotometrically at 530 nm for anthocyanins, at 350 nm for flavonols and at 280 nm for phenolic acids^15^.

The amounts of flavonols were calculated as quercetin 3-glucoside equivalents (ε 21877 Lm-1 mol-1, M. W. 464.38) and the amounts of phenolics as ferulic acid equivalents (ε 14700 Lm-1 mol-1, M.W. 194.18). The analyses were conducted three times for each genotype.

### Spectrophotometer determination of phlobaphenes

Phlobaphenes were extracted from individual seeds with one volume of concentrated HCl and four volumes of dimethyl sulfoxide (DMSO) added sequentially with vigorous vortexing after each addition, essentially as previously described^15^. Extracts were then centrifuged and cleared supernatants were diluted with methanol (20% final concentration). Phlobaphenes concentration was expressed as absorbance value at their λmax (510 nm) per 100 g of dry weight. The analyses were conducted on three replicates for each genotype.

### Enzyme immunoassay for the detection of fumonisins

For the determination of fumonisins concentration we used the kit “I’ screen FUMO” (Tecna®) according to manufacturer’s instructions.

Briefly: for each sample, 50 g of flour were extracted with methanol 70%, a dilution 1:20 of the extracts was loaded in the reaction plate and then the enzyme conjugate and the fumonisins antibody were added to each sample. After four washes the samples were incubated for 30 min with the developing solution and after the addition of the stop solution the absorbance was measured at 450 nm. The analyses were conducted three times for each genotype.

### Histological analysis

Seeds of Syn1c, Syn1r, Syn2c, Syn2r, Nsw, Nsr and *Ac* lines were imbibed in water overnight and longitudinally cut into halves with a scalpel.

To determine the pericarp thickness, images were taken and elaborated using a Leica MZ6 Stereoscope, equipped with 4x objective and 10x eyepieces (total magnification 40×) and the application software LAS V3.8. Statistical analysis was performed on at least 15 measurements of pericarp thickness for each genotype studied.

### Informatic tools

Microsoft Excel^®^ was used to collect data, PAST program (Paleontological Statistics, version 3.12) was used to perform statistical analysis.
